# Pandemic predisposing influence for feline lower urinary disorders

**DOI:** 10.3389/fvets.2025.1546288

**Published:** 2025-06-16

**Authors:** Carolina C. L. Paulino, André Meneses, Pedro Almeida, Paulo Dinis, Joana Tavares de Oliveira

**Affiliations:** ^1^Faculty of Veterinary Medicine of the Lusófona University, Lisboa University Center, Lisbon, Portugal; ^2^I-MVET – Investigação em Medicina Veterinária, Faculdade de Medicina Veterinária, Centro Universitário de Lisboa, Universidade Lusófona, Lisbon, Portugal; ^3^CECAV-Centro de Ciência Animal e Veterinária-Faculdade de Medicina Veterinária de Lisboa, Centro Universitário de Lisboa, Universidade Lusófona, Lisbon, Portugal; ^4^Faculty of Medicine of the University of Porto (FMUP), Porto, Portugal

**Keywords:** COVID-19, feline urinary disorders, home confinement, owner engagement, pandemic pet health, stress-related urinary conditions

## Abstract

Lower urinary tract signs (LUTS) in cats encompass a range of clinical symptoms, that may have been altered by the unique circumstances of confinement and increased human interaction during the lockdowns associated with the COVID-19 pandemic. The stress of confinement could exacerbate underlying behavioral issues, while increased caregiver presence might influence the expression of LUTS, potentially leading to either improved monitoring and early detection or increased stress-related symptoms. This study aimed to evaluate the impact of the COVID-19 pandemic on the presentation and outcomes of LUTS in cats by examining the potential influence of increased caregiver presence and confinement-related stress on clinical manifestations and disease progression. This retrospective study reviewed 298 cats with LUTS seen at a veterinary hospital from 2019 to 2021. Inclusion criteria required a complete set of examinations, including urinalysis, imaging, and urine culture. Cats were divided into a before-pandemic (BP) group and a during-pandemic (DP) group. Key variables, such as urethral obstruction, recurrence rates, and mortality, were statistically analyzed. The DP group demonstrated a statistically significant decrease in relapses (*p* < 0.001) and mortality (*p* < 0.05) compared to the BP group. Despite a rise in urethral obstructions in the DP group (*p* = 0.036), there was an overall reduction in adverse outcomes. BP cats were more likely to experience multiple episodes of LUTS than DP cats (*p* < 0.01). The findings suggest a positive impact of caregiver presence during the pandemic on LUTS outcomes, possibly due to improved monitoring and timely intervention. These insights underscore the potential benefits of increased caregiver engagement in managing feline LUTS.

## Introduction

Lower urinary tract signs (LUTS) in cats can arise from a variety of clinical conditions, including feline idiopathic cystitis (FIC), urolithiasis, urinary tract infection (UTI), neoplasia, and urethral obstructions. Among these, FIC remains a prevalent and challenging condition to manage, often diagnosed by exclusion when no specific etiology is identifiable (1). FIC can present as obstructive or non-obstructive, with the recurrent, non-obstructive form accounting for over 60% of cases (2). This idiopathic nature mirrors the human counterpart, bladder pain syndrome/interstitial cystitis, emphasizing the complexity of stress-related urinary conditions in both species. Research increasingly demonstrates that stress and environmental factors play a significant role in the manifestation of LUTS, particularly in idiopathic cases (3). Factors such as limited outdoor access, monotony in the indoor environment, and multi-cat household dynamics contribute to heightened stress levels, making cats particularly susceptible to FIC. Notably, McMillan et al. (4) observed that cats confined indoors without sufficient enrichment show elevated cortisol levels, indicative of a heightened stress response. Additionally, unpredictable environments, though sometimes beneficial for felines, can also be a source of stress, especially in sensitive or socially dependent cats.

The onset of the COVID-19 pandemic brought significant changes to pet ownership dynamics, with increased caregiver presence due to lockdowns and remote work arrangements. While Jezierski et al. (5) reported that caregiver presence led to heightened pet health awareness, there is debate over whether this increased attention might alleviate or exacerbate stress-related conditions such as LUTS. For example, Finstad et al. (6) found a reduction in adverse outcomes in cats whose caregivers were more engaged, while Kerley et al. (7) noted an increased incidence of urethral obstruction in households with heightened family activity during confinement. Understanding the impact of the pandemic on LUTS presentation and outcomes in cats is crucial, as it can help veterinarians adapt their approaches to diagnosis and treatment in light of the ongoing challenges posed by the pandemic. By evaluating these changes, we can better support the health and wellbeing of both cats and their caregivers moving forward.

This study seeks to explore the impact of pandemic-related environmental changes on LUTS. By comparing the outcomes of LUTS before and during the pandemic, this research aims to assess whether the increased caregiver presence acted as a mitigating or exacerbating factor in the progression and recurrence of LUTS in affected cats.

## Materials and methods

### Study design and population

This retrospective study included cats diagnosed with LUTS at a veterinary hospital between February 1, 2019, and January 30, 2021. Cases were divided into a before-pandemic (BP) group (February 1, 2019–January 30, 2020) and a during-pandemic (DP) group (February 1, 2020–January 30, 2021). These timeframes correspond with national confinement periods in Portugal (institutional approval number 2-2023).

### Inclusion and exclusion criteria

Cats were eligible for inclusion if they presented clinical signs of LUTS (e.g., dysuria, hematuria, periuria) and had complete diagnostic records, including physical examination, urinalysis [Urine Specific Gravity (USG), sediment, and culture], and imaging (radiography and/or ultrasound). Cats with comorbidities that could directly or indirectly influence LUTS, such as diabetes mellitus, hypertension, kidney disease, and neoplasia, were excluded. Cats that had been administered antibiotics or corticosteroids within 3 weeks before the episode were also excluded to prevent confounding effects.

### Data collection

Data collected included breed, sex, age, neuter status, type of diet, indoor/outdoor status, body condition score, cohabitants, clinical signs at presentation, and diagnostic outcomes (e.g., urethral obstruction, FIC, urolithiasis). Only the most severe episode per cat, based on clinical signs, creatinine and potassium levels, and confirmed obstruction, was considered for analysis. All clinical and diagnostic data collected for this study, including individual case details, are provided in Supplementary Tables 1, 2.

### Statistical analysis

Descriptive and inferential statistical analyses were conducted using IBM SPSS Statistics 22. Comparisons between the BP and DP groups on categorical variables (e.g., obstruction rates, mortality) were performed using the chi-square test, with significance set at *p* < 0.05.

## Results

### Study population and baseline characteristics

From a total of 862 cats screened, 298 met the inclusion criteria: 142 in the BP group and 156 in the DP group. Among the study population, 228 were male, and 70 were female, with ages ranging from 4 months to 20 years. Breed distribution included 92.3% non-pedigree cats, while the remaining were Siamese, Persian, Norwegian Forest, Scottish Fold, or British Shorthair.

### Clinical and diagnostic findings

The DP group showed a statistically significant reduction in both recurrence and mortality rates compared to the BP group. Specifically, relapse frequency was lower in the DP group (*p* < 0.001), with these cats more likely to experience a single episode of LUTS than those in the BP group (*p* < 0.01). Despite a higher rate of urethral obstruction (UO) in the DP group (*p* = 0.036), this group experienced a significant decrease in mortality (*p* < 0.05).

**Figure 1:**
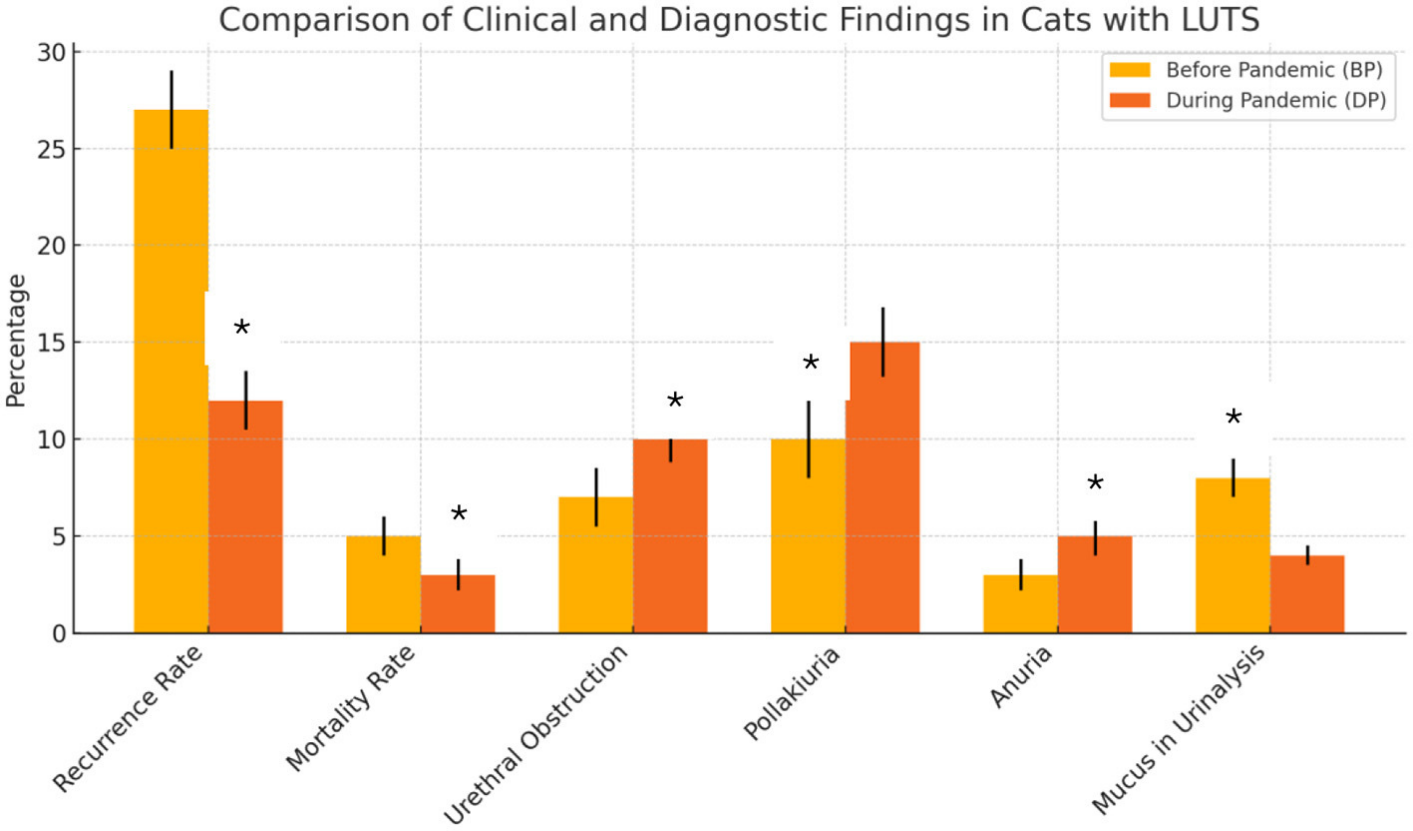
Comparison of clinical and diagnostic findings in cats with LUTS before and during the COVID-19 pandemic. Asterisks represent outcomes where the difference between groups was statistically significant (*p* < 0.05).

Despite anuria being more common in the DP group (*p* = 0.018), an increase in pollakiuria was noted in the BP group (*p* = 0.004), while cats in the BP group were more likely to present with mucus in the urinalysis (*p* = 0.02). No significant differences were observed between the two groups for most clinical signs (dysuria, hematuria, periuria, and stranguria). These data are expressed in Figure 1.

Detailed individual case data and statistical comparisons between the BP and DP groups are summarized in Supplementary Tables 1, 2.

### Diet and management factors

A shift in dietary management was observed, with non-prescription food more commonly fed to BP cats, while veterinary prescription diets were significantly more common in the DP group (*p* = 0.023). No differences were found between groups regarding dry or wet food preference (*p* = 0.496).

## Discussion

This study reveals that the COVID-19 pandemic had a measurable impact on LUTS outcomes, with a notable reduction in mortality, recurrence rates, and frequency of episodes in the DP group compared to the BP group. Despite an increased rate of UO in the DP group, these findings underscore the complex interplay between caregiver presence, environmental factors, and LUTS outcomes.

Ancillary diagnostic tests, including urinalysis, imaging, and urine culture, were critical in identifying LUTS etiologies. Chew et al. (2) and Buffington (1) emphasize the value of urinalysis and imaging in distinguishing FIC from urolithiasis, enabling precise diagnosis and management. In this study, urinalysis revealed a high incidence of crystalluria in urolithiasis cases, while lipiduria was more commonly associated with FIC (*p* < 0.001). These findings align with Stella et al. (3), who noted that stress-exacerbated urinary signs can be differentiated through urinalysis findings. Imaging, as reported by McMillan et al. (4), is particularly valuable for identifying structural abnormalities, the conclusion this study confirmed in cases of obstructive LUTS.

The reduction in adverse outcomes among DP cats may be attributed to increased caregiver vigilance and engagement during lockdowns. Finstad et al. (6) found that owner-pet interactions positively affected pets' chronic conditions, such as LUTS, by enhancing health monitoring and enabling timely intervention. Jezierski et al. (5) similarly noted that lockdown conditions allowed caregivers to observe early signs of illness, leading to prompt veterinary consultations. This increased caregiver awareness, combined with the observed shift toward prescription diets in the DP group, may have also played a preventive role. As Buffington (1) demonstrated, dietary management is essential for mitigating the recurrence of FIC and urolithiasis.

Nevertheless, the increased UO rate in the DP group calls for a nuanced interpretation. While closer observation may have facilitated early detection, confinement stress may have worsened LUTS in some cats, particularly those sensitive to environmental changes. Research by Kerley et al. (7) noted an increase in UO among cats in high-activity households during the pandemic, indicating that greater family presence and potential disruptions in routine could heighten stress responses in some felines. This finding aligns with Stella et al. (3), who suggested that environmental stressors, particularly in multi-pet or active households, are risk factors for LUTS in cats predisposed to stress-related conditions.

Conversely, Jackson et al. (8) observed that some cats adapted well, or even thrived from, the increased interaction, underscoring the variability in feline responses to confinement. This resilience among certain cats highlights the need to individualize stress management approaches. Stella et al. (3) emphasize the importance of tailored environmental enrichment to support each cat's unique stress tolerance. In some cases, confinement may have reinforced the human-cat bond, which is an essential factor in feline welfare. This is supported by studies from both Finstad et al. (6) and Jezierski et al. (5), which found that positive caregiver interactions could mitigate some stress-induced health risks.

Studies by McMillan et al. (4) and Kerley et al. (7) emphasize the influence of individual household dynamics, suggesting that granular data on environmental changes would further clarify the relationship between confinement and LUTS. Future studies should consider a more detailed examination of household factors to fully capture the impact of caregiver behaviors and confinement-specific variables on feline health outcomes.

The findings in this study underscore the importance of educating caregivers on managing feline stress and the benefits of attentive monitoring, particularly for cats with recurrent LUTS. As Buffington (1) and Stella et al. (3) propose, maintaining an enriched environment and consistent routines can play a vital role in improving health outcomes for cats with stress-exacerbated conditions. Future research into the biopsychosocial effects of caregiver engagement on pet health will be essential to understand the broader implications of changes in environmental conditions on feline LUTS outcomes.

This study has several limitations. First, the retrospective design limits control over confounding variables, such as variations in veterinary care access during the pandemic, which may have influenced the observed outcomes. The use of historical data also restricted our ability to directly follow up on cats with recurrent LUTS episodes, limiting insight into longer-term management and recurrence trends. Additionally, the study did not account for the potential impact of individual caregiver behaviors or specific environmental changes on feline stress levels, as these factors could not be ascertained from the available clinical data. Finally, the variability in responses to confinement across different households suggests that future research should consider a more granular approach, examining individual household factors and their effects on feline LUTS.

## Conclusion

The findings from this study highlight a significant impact of the COVID-19 pandemic lockdowns on the presentation and outcomes of LUTS in cats. Despite a rise in urethral obstructions in the DP group, the overall reduction in adverse outcomes, including lower recurrence and mortality rates, suggests that increased caregiver presence may have played a critical role in the early detection and management of feline LUTS in this context. These results underscore the importance of consistent caregiver involvement and adherence to dietary recommendations in mitigating chronic LUTS conditions and preventing acute-on chronic flare-ups. Future studies should explore the specific aspects of caregiver-pet interactions that contribute most to improved health outcomes, particularly under stress-related circumstances.

## Data Availability

The datasets presented in this study can be found in online repositories. The names of the repository/repositories and accession number(s) can be found at: https://recil.ulusofona.pt/items/993197ca-a8bd-4130-9cc5-4fa28e38ec9b ReCiL-Repositório Científico Lusófona https://recil.ensinolusofona.pt/.
